# Full-color enhanced second harmonic generation using rainbow trapping in ultrathin hyperbolic metamaterials

**DOI:** 10.1038/s41467-021-26818-3

**Published:** 2021-11-05

**Authors:** Junhao Li, Guangwei Hu, Lina Shi, Nan He, Daqian Li, Qiuyu Shang, Qing Zhang, Huange Fu, Linlin Zhou, Wei Xiong, Jianguo Guan, Jian Wang, Sailing He, Lin Chen

**Affiliations:** 1 Wuhan National Laboratory for Optoelectronics and School of Optical and Electronic Information, Huazhong University of Science and Technology, Wuhan, 430074 China; 2 Department of Electrical and Computer Engineering, National University of Singapore, 4 Engineering Drive 3, Singapore, 117583 Singapore; 3 Key Laboratory of Microelectronic Devices and Integrated Technology, Institute of Microelectronics, Chinese Academy of Sciences, Beijing, 100029 China; 4 Centre for Optical and Electromagnetic Research, Zhejiang Provincial Key Laboratory for Sensing Technologies, JORCEP, Zhejiang University, Hangzhou, 310058 China; 5 School of Materials Science and Engineering, Peking University, Beijing, 100871 China; 6 State Key Laboratory of Advanced Technology for Materials Synthesis and Processing, Wuhan University of Technology, Wuhan, 430074 China; 7 Department of Electromagnetic Engineering, School of Electrical Engineering, Royal Institute of Technology, S-100 44 Stockholm, Sweden

**Keywords:** Metamaterials, Nonlinear optics

## Abstract

Metasurfaces have provided a promising approach to enhance the nonlinearity at subwavelength scale, but usually suffer from a narrow bandwidth as imposed by sharp resonant features. Here, we counterintuitively report a broadband, enhanced second-harmonic generation, in nanopatterned hyperbolic metamaterials. The nanopatterning allows the direct access of the mode with large momentum, rendering the rainbow light trapping, i.e. slow light in a broad frequency, and thus enhancing the local field intensity for boosted nonlinear light-matter interactions. For a proof-of-concept demonstration, we fabricated a nanostructured Au/ZnO multilayer, and enhanced second harmonic generation can be observed within the visible wavelength range (400-650 nm). The enhancement factor is over 50 within the wavelength range of 470-650 nm, and a maximum conversion efficiency of 1.13×10^−6^ is obtained with a pump power of only 8.80 mW. Our results herein offer an effective and robust approach towards the broadband metasurface-based nonlinear devices for various important technologies.

## Abstract (web summary)

Though metamaterials enhance nonlinear light-matter interactions due to their resonant features, these materials typically show a narrow spectral bandwidth. Here, the authors report broadband enhanced second-harmonic generation in patterned multilayer hyperbolic metamaterial arrays.

## Introduction

Nonlinear optics deals with light interaction with materials beyond of the simple description of linear relationships between the induced polarization and incident field, which has widespread applications in laser technology^1^, microscopy^2^, and material science^3^. As the high-order term between materials’ polarizability and pumping^4^, the nonlinear optical responses of crystals are usually weak and thus require the stringent phase-matching condition or quasi-phase-matching condition, and sufficient light-matter interaction length, to boost the nonlinear light generation. Consequently, the resultant nonlinear devices developed accordingly are bulky, which is against highly integrated on-chip photonic technologies. Thus, searching more efficient nonlinear materials, such as emerging two-dimensional nanomaterials^5,6^, or the new physical mechanism^7–9^ to boost materials’ nonlinearity becomes an important branch in modern optics.

Recent studies have shown great promise in metamaterials and metasurfaces to enhance nonlinear light-matter interactions within subwavelength nanostructures, remarkably relaxing phase-matching conditions^10–12^ and developing multifunctional nonlinear photonic components in the nanoscale. Plasmonic metasurfaces can squeeze light into a deep subwavelength volume and thus greatly enhance nonlinear harmonic generation, but the Ohmic loss and the weak penetration of the field inside the metal significantly limits the conversion efficiency^11–15^. In that sense, all-dielectric low-loss nanostructures are widely explored, as high-quality Mie resonances supported therein can compress the pump light to excite nonlinear processes and meanwhile efficiently extract nonlinear signals serving as the nanoantenna^16–20^. Other approaches such as hybrid nonlinear metasurfaces combining a resonant metallic structure with another dielectric or novel nonlinear material have also been proposed, further boosting frequency conversion processes^9,21–26^. Nevertheless, those approaches based on high-quality resonance intrinsically suffer from the narrow bandwidth, which limits broadband nonlinear applications.

In a rather different context, hyperbolic metamaterials (HMMs), with an extreme anisotropy supporting effective permittivities along different directions of different signs, have enabled a variety of broadband applications including indefinite cavities^27^, enhanced spontaneous emission^28–30^, hyperlens^31^, optical absorption^32,33^, and wavefront manipulation^34^. The physical origin comes from their exotic hyperbolic dispersions, with very large momentum compared to free-space photons, to support boosted light-matter interactions, and does not require the resonances in principle. Although efforts have been made to enhance optical nonlinearities in HMMs such as metal nanorod arrays and a nonlinear dielectric core wrapped by spherical metal/dielectric multilayer cavities^35–39^, the narrow spectral bandwidth is still inevitable due to the introduced resonances to satisfy stringent phase-matching conditions.

**Fig. 1:**
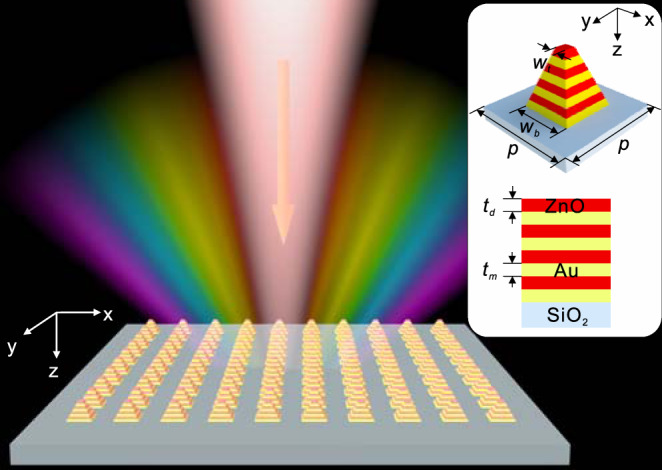
Schematic of a full-color second harmonic generator made of TP-HMMs. The inset is an enlarged view of its unit cell. The alternating metal and dielectric materials are Au and ZnO, with the thickness being marked as *t*_m_ and *t*_d_, respectively. The HMM width is linearly increased from *w*_t_ to *w*_b_, and the periods along the *x*- and *y*-directions are equal and are denoted as *p*.

In this study, we demonstrated the broadband, covering nearly full visible frequency, enhanced second harmonic generation (SHG) in patterned HMM arrays comprising metal/dielectric multilayer (see Fig. 1). Our results are majorly attributed to the giant field localization due to the broadband and efficient coupling of light at fundamental frequencies, thanks to the frequency-dependent spatially localized slow light (termed as “rainbow trapping”)^40–44^. We also experimentally observed significantly enhanced SHG within a pump wavelength range of 800–1300 nm, i.e., full-color visible SHG photographs within the wavelength range of 400–650 nm. Our reports here open a new avenue towards the broadband nonlinear functional devices.

## Results and discussion

### Working principle

Figure 1 schematically shows our design, a full-color second harmonic generator made of two-dimensional (2D) taper-patterned HMM (TP-HMM) arrays, comprising alternating Au and ZnO layers. It is noteworthy that ZnO without inversion symmetry is a dielectric that allows SHG. Here, SHG signals are generated and collected at the reflection side when normally incident light pumps the structures and thus induces strong field localization in the ZnO layer at fundamental frequencies. In a general remark, the broadband field enhancement and hence broadband SHG are achieved by designing an array of HMMs pillars with the graded width, i.e., the TP-HMM unit cells with the width from *w*_b_ (the width at the bottom) to *w*_t_ (the width at the top) as described in the inset of Fig. 1.

**Fig. 2:**
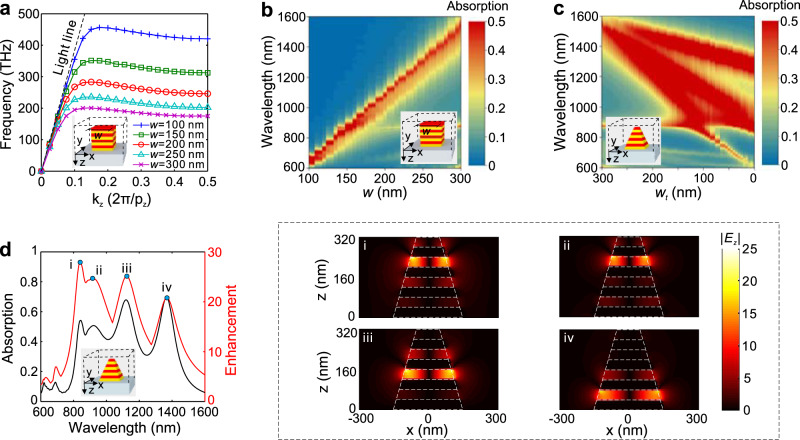
Rainbow trapping in TP-HMMs. **a** Dispersion curves of the SSP mode in U-HMMs with widths of *w* = 100, 150, 200, 250, and 300 nm. The propagation constant along the *z*-axis, ${k}_{z}$, is normalized to $2{{{{{\rm{\pi }}}}}}/{p}_{z}$, with *p*_z_ = *t*_m_ = *t*_d_. **b** Absorption for U-HMM with a varying *w*. **c** Absorption for TP-HMM with a varying top width of *w*_t_ = 300–0 nm and a constant bottom width of *w*_b_ = 300 nm. **d** Absorption and near-field enhancement of ${E}_{z}$ for the TP-HMM with. (i–iv) Field distributions of $|{E}_{z}|$ (normalized to the incident light) in the *x*–*z* plane of the TP-HMM at absorption peaks of 840, 915, 1123, and 1369 nm. In **a**–**d**, the geometrical parameters are set at *t*_m_ = 40 nm, *t*_d_ = 40 nm, and *p* = 600 nm. In **b**–**d**, four pairs of Au/ZnO layers are used and the incidence is an *x*-polarized normally incident plane wave. The permittivities of Au and SiO_2_ are from the Palik model^50^, and the permittivity of ZnO is from ref. ^51^. All the simulations are conducted by finite difference time domain (FDTD) with the commercial software Lumerical FDTD Solutions.

To explain the underlying working principle, we start from the untapered but patterned HMM (U-HMM) pillar arrays, i.e., *w*_b_ = *w*_t_ = *w* (see the inset in Fig. 2a). As demonstrated, a spoof surface plasmon mode at such U-HMM pattern can be supported, with a flatband feature inducing the significantly reduced group velocity, so-called “slow-light trapping” effect (see Fig. 2a). Importantly, such effect can be flexibly controlled by the HMM geometry^34^ (see Supplementary Fig. S1 and theoretical derivations in Supplementary Note 1), exhibiting a continuously decreased slow-light frequency with respect to the increasing width of pillar. The fundamental reason behind is the constructive interference of plasmonic modes reflected by the sidewalls of the pillar, rendering a Fabry–Perot cavity, which also strongly depends on the width of the pillar^34^. However, the U-HMM is intrinsically narrowband, which would only support the remarkable absorption (Fig. 2b) and near-field enhancement (see Fig. S5a in Supplementary Note 2) at a singular frequency, unsuitable for the broadband optical components.

Therefore, it is important to introduce our tapered patterns to realize the broadband performance. As shown in Fig. 2c, the broadband absorption exists in TP-HMM with a cross-section width varying continuously, which is also associated with near-field enhancement (see Supplementary Fig. S5b). Here, we keep *w*_b_ = 300 nm and vary *w*_t_ from 300 nm (e.g., U-HMM) to 0 nm (e.g., the triangle pyramid structure), from which we see the emergence of more resonances that essentially give rise to broadband absorptions. This could be intuitively pictured as the TP-HMM pillar consists of many U-HMM pillars with a continuously varying width and thus inherits the slow-light effect at different frequencies supported therein, which results in so-called “rainbow light trapping” effect. Specifically, for an optimal structure with *w*_t_ = 100 nm, we show its absorption and near-field enhancement characteristics in Fig. 2d (see Supplementary Fig. S5c, d for more details of near-field enhancement). The broadband near-field enhancement and the efficient coupling to the tapered mode from free-space pumping can be clearly observed from the simulated absorption spectrum, implying the important reason for enhanced SHG as will be demonstrated. The multiple peaks of absorption and enhancement spectrums can find the root of width-dependent slow-light effect, which can be further demonstrated by Supplementary Fig. S6 (see Supplementary Fig. S6 in Supplementary Note 2 for absorption and enhancement with varying Au/ZnO pairs). The rainbow light trapping and the field enhancement supported in TP-HMM at fundamental frequencies can be seen from Fig. 2d(i–iv) and Supplementary Fig. S7 in Supplementary Note 2, which will be exploited to boost SHG.

Before closing this section, we remark on several other important features. First, the C6 symmetry and Kleinman’s symmetry conditions on ZnO layers lead to two independent nonzero components of the second-order susceptibility tensor: ${\chi }_{xzx}={\chi }_{yzy}={\chi }_{xxz}={\chi }_{yyz}={\chi }_{zxx}={\chi }_{zyy}$ and ${\chi }_{zzz}$^45^. We see that the dominant electric field component at fundamental frequencies in ZnO layers is z-component, i.e., *E*_*z*_. Therefore, SHG mainly comes from ${\chi }_{zzz}$, whereas all contributions from other component can be safely ignored. However, this introduces a problem, as the induced nonlinear field will be dominant nonradiative *E*_*z*_ component. Fortunately, our patterned HMM arrays can brighten the *z*-oriented dipole at second harmonic frequencies and enhance their radiative rates by up to 38 times as compared to the unpatterned area in the Au/ZnO multilayer (termed as “unpatterned area,” see radiative enhancement in Supplementary Fig. S8 of Supplementary Note 2), which is another unique advantage of our structures^46^.

Second, we can further improve the enhancement of broad spectrum in Fig. 2d, by choosing optimized pairs of alternating layers in our TP-HMM (see more numerical results in Supplementary Fig. S6 of Supplementary Note 2). Nevertheless, we provide an alternative avenue to further enhance the broadband performance by designing dual TP-HMM pillars with different geometries in a unit cell, while keeping only four pairs of Au/ZnO layers without complicating the required fabrication when more Au/ZnO pairs are introduced. In such a dual-pillar TP-HMM, the light will be localized in one of HMM pillars, depending on the incident wavelength^33^. As a result, the near-field intensity can be increased, in contrast with the single-pillar TP-HMM array, while the working bandwidth is almost the same. At the pump wavelengths to be tested (800, 900, 1000, 1100, 1200, and 1300 nm), the dual-pillar TP-HMM array design offers even stronger field enhancement of $|{E}_{z}|$ ranging from 15.5 to 35.0 (see Supplementary Fig. S9 of Supplementary Note 2), superior over the single-pillar TP-HMM with the near-field enhancement factor ranging from 14.0 to 24.4. Such a dual-pillar TP-HMM array design offers even stronger field enhancement, which is therefore adopted in our experiment.

### Experimental verification of broadband and efficient SHG

**Fig. 3:**
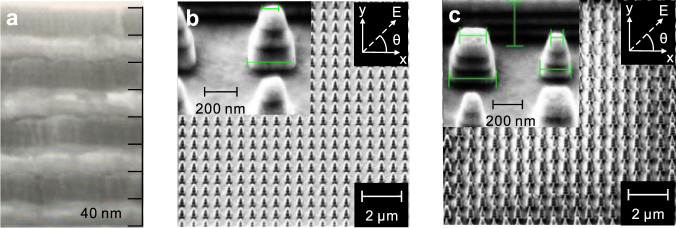
The fabricated TP-HMMs. **a** SEM image of the vertical cross-section of the unpatterned Au/ZnO multilayer. **b** SEM image of the single-pillar TP-HMM. **c** SEM image of the dual-pillar TP-HMM.

**Fig. 4:**
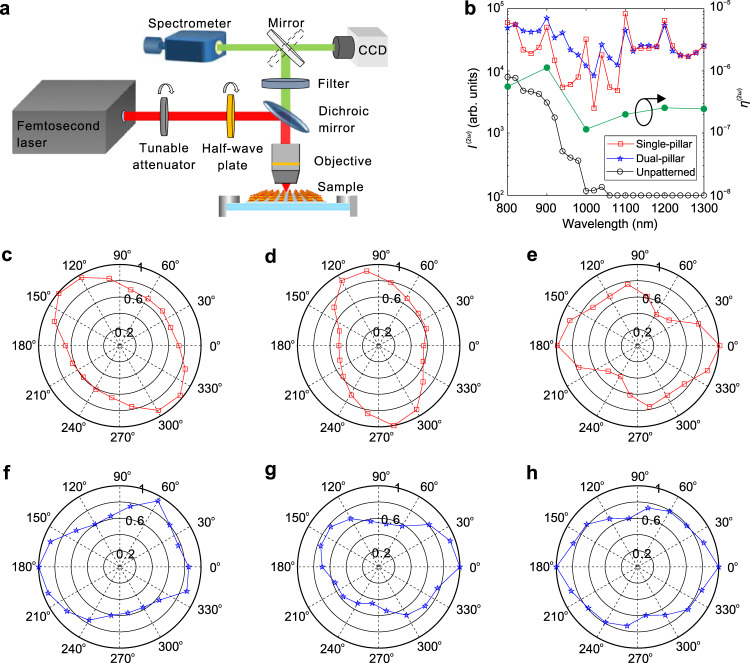
Testing setup and broadband SHG. **a** The schematic of home-made testing setup for the SHG spectrograph and microscopy. **b** ${I}^{(2\omega )}$ and ${\eta }^{(2\omega )}$ as functions of the pump wavelength for single-pillar and dual-pillar TP-HMMs, with the pump light being *x*-polarized and normally incident. **c**–**h** Normalized ${I}^{(2\omega )}$ for single-pillar (**c**–**e**) and dual-pillar (**f**–**h**) TP-HMMs as a function of the polarization angle, *θ*, of the linearly polarized pump light at the central wavelength of 800 (**c**, **f**), 1000 (**d**, **g**), and 1200 nm (**e**, **h**). The angular axis indicates *θ* and the radial axis indicates the normalized (to the maximum) ${I}^{(2\omega )}$.

The sample fabrication process starts with four pairs of Au/ZnO layers alternately deposited on the silica substrate by using magnetron sputtering (Fig. 3a). Then, the required tapered patterns are formed by using a focused ion beam (FIB) milling. The fabricated single-pillar TP-HMM and dual-pillar TP-HMM pillar arrays are presented in Fig. 3b, c, respectively. The SHG measurement is experimentally implemented with the home-made testing setup, as schematically shown in Fig. 4a. The measured SHG intensity, ${I}^{(2\omega )}$, as a function of the fundamental wavelength is presented in Fig. 4b, where ${I}^{(2\omega )}$ is a normalized value extracted from the spectrometer^15,16,19,25^. ${I}^{(2\omega )}$ is over 6000 in the entire pump wavelength range of 800–1300 nm for the 2D dual-pillar TP-HMM, showing better SHG performance over the 2D single-pillar TP-HMM, where ${I}^{(2\omega )} > 2500$ lies in the entire wavelength range of 800–1300 nm. In addition, the SHG intensity of the dual-pillar TP-HMM is higher than that of the single-pillar TP-HMM at most of pump wavelengths (20 out of 26 wavelength points), associated to the stronger near-field enhancement by using the double-sized tapered HMMs. For the other six points, the single pillar showed higher SHG intensity than the dual pillar; due to this, the multiple peaks and valleys of enhancement spectrums for the single-pillar and dual-pillar TP-HMMs are not at the same wavelengths. TP-HMMs show significantly enhanced SHG intensity over the unpatterned area. The SHG enhancement factor (EF) is defined as the ratio of SHG intensity when the incident light is focused onto the patterned sample and the unpatterned area. The resultant EF is over 50 in 1100–1300 nm for the single-pillar TP-HMM, whereas that for the dual-pillar TP-HMM is over 50 in the pump wavelength range of 940–1300 nm and reaches the maximum value of about 535 at 1200 nm (see Supplementary Fig. S10 of Supplementary Note 2).

Supplementary Fig. S11 shows the SHG response on the pump power for the TP-HMMs, indicating that the SHG intensity is nearly a quadratic function of the pump power^9,12^. Figure 4c–f verifies that the TP-HMMs present little polarization dependence, as the SHG intensity varies gently with the polarization angle *θ*. This is expected as the single-pillar TP-HMM carries the C4 rotation symmetry with respect to the *z*-axis and the light is pumped normally (see Supplementary Fig. S12 of Supplementary Note 2). For dual-pillar TP-HMM, the interleaved TP-HMM pillars with different size renders the C4 rotation symmetric arrangement. Therefore, those two devices are very useful for polarization-independent nonlinear optical elements. Nevertheless, for some specific applications when polarization-dependent nonlinear optical responses are required, we can resort to one-dimensional TP-HMM as shown in Supplementary Figs. S2–S4 of Supplementary Note 1.

The SHG signals with different pump wavelengths are demonstrated by the micrographs captured by the charge-coupled device (CCD) camera, where full-color SHG signals in the visible region are clearly presented in Supplementary Fig. S13 of Supplementary Note 2. In nonlinear optical processes, the SHG conversion efficiency is a key factor for practical applications. Here the SHG conversion efficiency, ${\eta }^{(2\omega )}$, is defined as ${\eta }^{(2\omega )}={P}^{(2\omega )}/{P}^{(\omega )}$^39^, where ${P}^{(2\omega )}$ is the light power of the SHG signal and ${P}^{(\omega )}$ is the pump power. With ${P}^{(\omega )}$ measured by a light power meter and ${P}^{(2\omega )}$ extracted from the SHG micrographs recorded by the CCD, ${\eta }^{(2\omega )}$ of the dual-pillar TP-HMM can be estimated and is presented in Fig. 4b (see more details in Supplementary Note 3). The maximum ${\eta }^{(2\omega )}$ of 1.13 × 10^−6^ is obtained with a pump wavelength of 900 nm and pump power of 8.80 mW for the dual-pillar TP-HMM. The 10 dB bandwidth, defined as the wavelength range with ${\eta }^{(2\omega )}$ >10% of its maximum, covers the entire pump wavelength range of 800–1300 nm. A comprehensive comparison of the device performance with some typical reports on metasurface-enabled SHG enhancement in the visible has been summarized in Table 1. The previous studies on nonlinear metasurfaces rely on the resonant structures so that the SHG conversion efficiencies reduce sharply once the working frequencies deviate from the resonant frequencies. The HMM proposal here has significantly broadened the working bandwidth, whereas the SHG conversion efficiency is comparably high.

**Table 1:** Performance comparison.

References	EF	${\eta }^{(2\omega )}$	Peak pump intensity (GW/cm^2^)	Average pump power (mW)	10 dB Bandwidth (nm)
^15^	NA	5.4 × 10^−10^	NA	150	N/A
^16^	10,000	2 × 10^−5^	3.4	11.4	~1010–1070
^19^	∼10,000	6 × 10^−5^	3.4	11.4	~970–1000
^22^	1700	∼3 × 10^−7^	60	NA	~780–806
^23^	NA	NA	0.3	47.1	~750–855
^25^	300	2 × 10^−6^	<63.7	0.8	~770–860
Our work	535	1.13 × 10^−6^	~8.73	8.80	800–1300

To sum up, we have demonstrated that tapered HMM nanostructure arrays enable broadband, enhanced field localizations due to the rainbow trapping effect, thus significantly boosting the SHG conversion efficiencies over a wide spectral bandwidth. Full-color SHG within the wavelength range of 400–650 nm has been experimentally observed, superior over the previous metasurface-based nonlinear devices. A maximum SHG conversion efficiency of 1.13 × 10^−6^ is obtained with an 8.80 mW pump at the wavelength of 900 nm. Our results here may solve the long-standing bandwidth issue with nonlinear metasurfaces and advance new technically feasible applications. Future work could use our proposals to devise broadband nonlinear optical devices and could even tailor the naturally born hyperbolic materials such as van der Waal structured nanomaterials for other broadband devices^47–49^.

## Methods

### FIB etching

In the FIB process, a constant current of Ga+ ions (with an electric current of 120 pA and a dwell time of 10 μs) is focused onto the multilayer under normal incidence and the position of the beam can be adjusted by a lithography system. Grayscale bitmaps are also used to precisely determine the tapered angle. The etching depth ratio between different pixels is controlled by setting the grayscale intensity in the bitmaps, whereas the absolute etching depth by setting the overall dose.

### SHG measurement

A femtosecond mode-locked Ti:sapphire laser (Coherent, Chameleon Discovery, 100 fs pulse width, 80 MHz repetition rate) is used as the light source that generates linearly polarized laser beam with the working wavelength tunable from 680 to 1300 nm and a narrow full width at half maximum of about 10 nm. A tunable attenuator is used to tune the pump power onto the sample; a half-wave plate is used to rotate the polarization angle to check the polarization response of the HMM samples. Reflected by a short-pass dichroic mirror (Thorlabs, DMSP750B), the incident light is focused onto the sample by a × 40 objective (Olympus, LUCPLFLN40X, NA = 0.6). The SHG signals and reflected pump light on the reflection path are collected with the same objective. To guarantee pure SHG signals reach the spectrometer (Princeton Instrument, Isoplane-320) and the CCD camera (Olympus, DP22), a short-pass dichroic mirror (Thorlabs, DMSP750B) and a short-pass filter (Thorlabs, FES0700) are inserted along the reflection path to filter the reflected pump light.

## Supplementary information

Supplementary Information

## Data Availability

All relevant data that support the findings of this study are available from the corresponding authors upon reasonable request.

## Code availability

The code that supports the plots within this study is available from the corresponding authors upon reasonable request.
