# Supplementary material for: Full-color enhanced second harmonic generation using rainbow trapping in ultrathin hyperbolic metamaterials
Source: Nat Commun. 2021 Nov 5;12:6425. doi: 10.1038/s41467-021-26818-3 (PMC8571340; doi:10.1038/s41467-021-26818-3)
Supplement: Supplementary file 1 — Supplementary Information [file 41467_2021_26818_MOESM1_ESM.pdf]

Supplementary Information for:

Full-Color Enhanced Second Harmonic Generation Using Rainbow Trapping in Ultrathin Hyperbolic Metamaterials

Junhao Li,[1] Guangwei Hu,[2] Lina Shi,[5] Nan He,[3] Daqian Li,[1] Qiuyu Shang,[6] Qing Zhang,[6,*] Huange Fu,[1] Linlin Zhou,[1] Wei Xiong,[1,*] Jianguo Guan,[7] Jian Wang,[1] Sailing He,[3,4] and Lin Chen[1,*]

[1]Wuhan National Laboratory for Optoelectronics and School of Optical and Electronic Information, Huazhong University of Science and Technology, Wuhan 430074, China.

[2]Department of Electrical and Computer Engineering, National University of Singapore, 4 Engineering Drive 3, Singapore 117583.

[3]Centre for Optical and Electromagnetic Research, Zhejiang Provincial Key Laboratory for Sensing Technologies, JORCEP, Zhejiang University, Hangzhou 310058, China.

[4]Department of Electromagnetic Engineering, School of Electrical Engineering, Royal Institute of Technology, S-100 44 Stockholm, Sweden.

[5]Key Laboratory of Microelectronic Devices & Integrated Technology, Institute of Microelectronics, Chinese Academy of Sciences, Beijing 100029, China.

[6]School of Materials Science and Engineering, Peking University, Beijing 100871, China.

[7]State Key Laboratory of Advanced Technology for Materials Synthesis and Processing, Wuhan University of Technology, Wuhan 430074, China.

These authors contributed equally: Junhao Li, Guangwei Hu, Lina Shi

Email: q_zhang@pku.edu.cn; weixiong@hust.edu.cn; chen.lin@mail.hust.edu.cn

Note 1: SHG with 1D HMMs

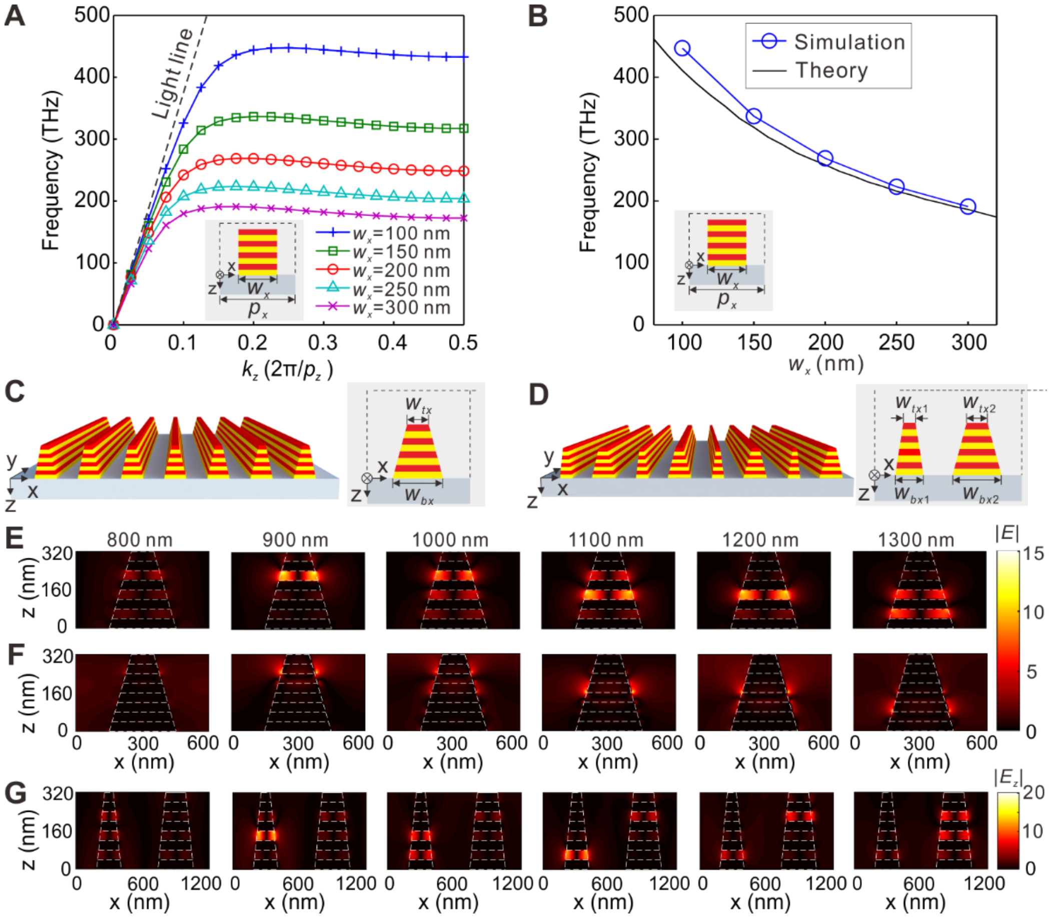

Fig. S1 Rainbow trapping in 1D TP-HMMs. a Dispersion curves for the 1D uniform HMM with different widths of w = 100, 150, 200, 250, and 300 nm. **b** Slow-light frequency as a function of w. The theoretical curve is obtained with Equation S2. **c** Schematic of the 1D single-pillar TP-HMM. **d** Schematic of the 1D dual-pillar TP-HMM. The insets in (a-d) are vertically cross-sectional views of the unit cells. **e** Field distributions of $|E_z|$ for the 1D single-pillar TP-HMM. **f** Field distributions of $|E_x|$ for the 1D single-pillar TP-HMM. **g** Field distributions of $|E_z|$ for the 1D dual-pillar TP-HMM. In (e-g), the TP-HMMs are under x-polarized normal incidence at the wavelengths of 800, 900, 1000, 1100, 1200, and 1300 nm. For the 1D single-pillar TP-HMM, $w_{tx} = 100$ nm, $w_{bx} = 300$ nm, and the lattice constant along the x direction is 600 nm. For the 1D dual-pillar TP-HMM, $w_{tx1} = 80$ nm, $w_{bx1} = 220$ nm, $w_{tx2} = 180$ nm, $w_{bx2} = 320$ nm, and the lattice constant along the x direction is 1200 nm.

According to the effective medium theory, the permittivity tensor of HMM can be approximately written as

$$
\begin{cases} \varepsilon_{\perp} = \dfrac{\varepsilon_m t_m + \varepsilon_d t_d}{t_m + t_d} \\ \varepsilon_{\parallel} = \dfrac{t_m + t_d}{t_m / \varepsilon_m + t_d / \varepsilon_d} \end{cases} \tag{S1}
$$

where ε_m and ε_d are the permittivities of the metal (Au) and dielectric (ZnO) materials, respectively. Then the propagation constant, k_z, can be calculated by[A1]

$$
\frac{\exp(-i\gamma_2 w) - \exp[\gamma_1 (P-w)]}{\exp(i\gamma_2 w) - \exp[\gamma_1 (P-w)]} = \frac{\exp(-i\gamma_2 w) - \exp[-\gamma_1 (P-w)]}{\exp(i\gamma_2 w) - \exp[-\gamma_1 (P-w)]} \left(\frac{\gamma_2 \varepsilon_1 + i\gamma_1 \varepsilon_z}{\gamma_2 \varepsilon_1 + i\gamma_1 \varepsilon_z} \right)^2 \tag{S2}
$$

where $\gamma_1 = \sqrt{k_z^2 - k_0^2 \varepsilon_1}$, $\gamma_2 = \sqrt{k_0^2 \varepsilon_z - \varepsilon_z {k_z}^2 / \varepsilon_x}$, and the vacuum wave vector $k_0 = \omega / c$. We have calculated the slow-light frequencies of 1D TP-HMMs with different widths by using Equation S2 (Fig. S1b).

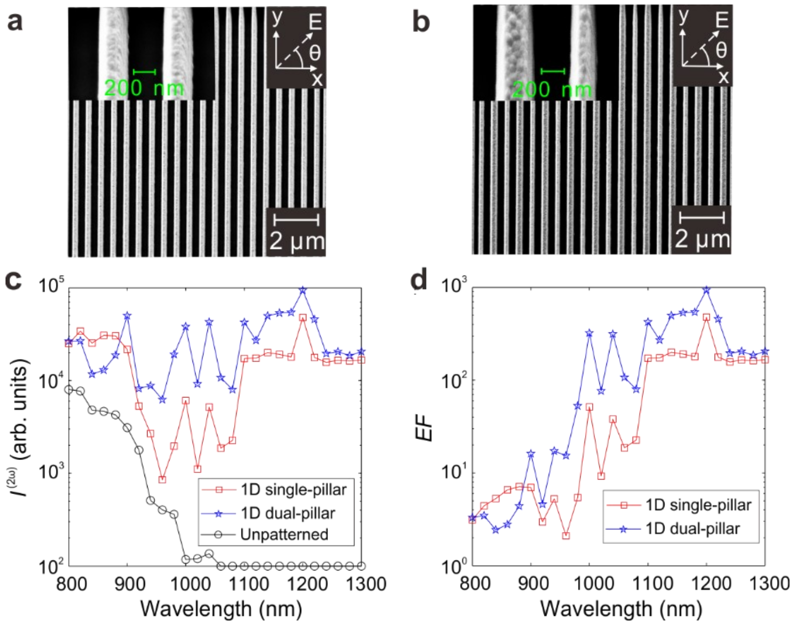

Fig. S2 Experimental Results for 1D TP-HMMs. a SEM image of the 1D single-pillar TP-HMM. **b** SEM image of the 1D dual-pillar TP-HMM. **c** $I^{(2\omega)}$ as a function of the pump wavelength under x-polarized normal incidence. **d** *EF* as a function of the pump wavelength under x-polarized normal incidence.

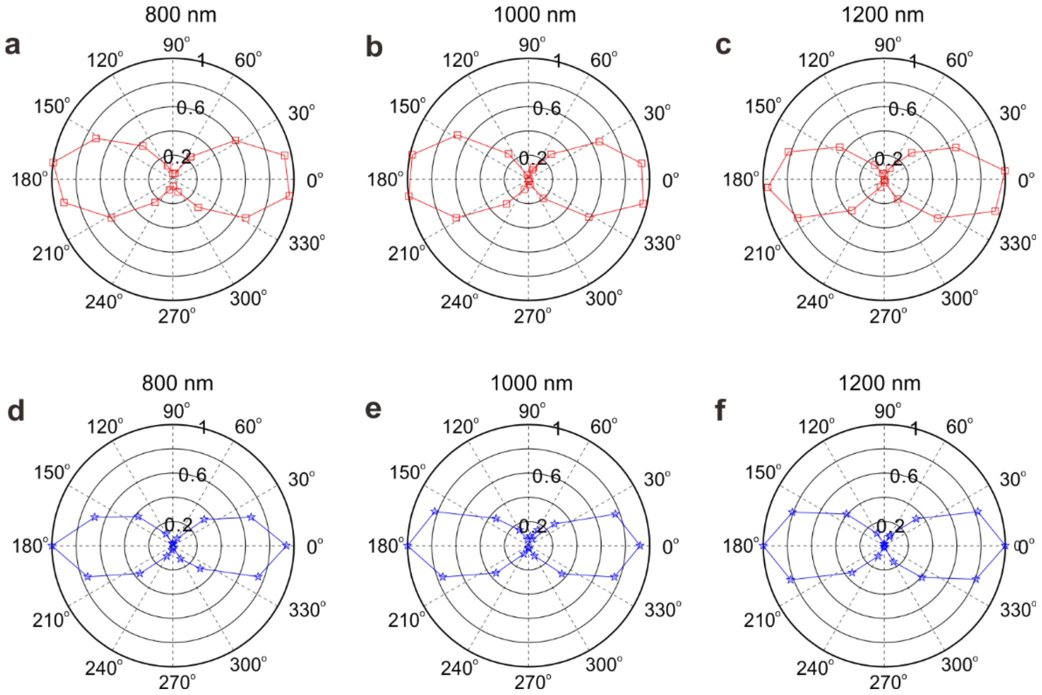

Fig. S3 Measured polarization responses for the 1D TP-HMMs. Normalized $I^{(2\omega)}$ for the 1D single-pilar (**a**-**b**) and dual-pillar TP-HMMs (**d**-**f**) as a function of the polarization angle, θ, with a pump wavelength of 800 (a, d), 1000 (b, e), and 1200 nm (c, f), respectively. The angular axis indicates the polarization angle, and the radial axis indicates the normalized (to the maximum) $I^{(2\omega)}$.

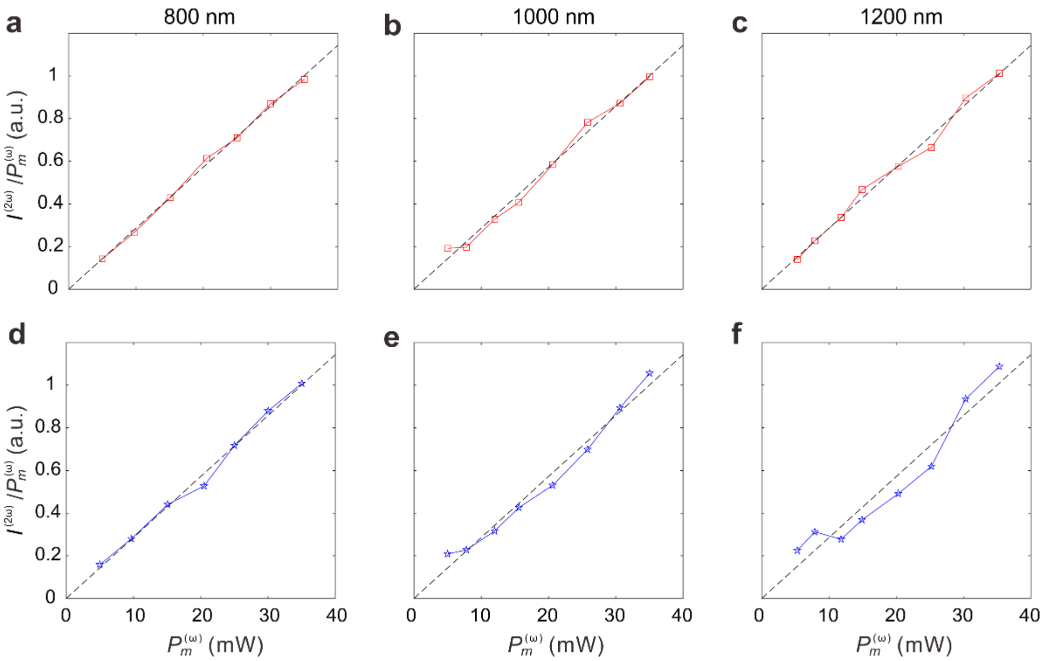

Fig. S4 Measured pump power responses for the 1D TP-HMMs. Normalized $I^{(2\omega)}/P_m^{(\omega)}$ for the 1D single-pilar (**a**-**c**) and dual-pillar TP-HMMs (**d**-**f**) as a function of $P_m^{(\omega)}$ with a pump wavelength of 800 (a, d), 1000 (b, e), and 1200 nm (c, f), respectively. The black dashed lines are the fitting data.

[A1] Ji, D. et al. Broadband absorption engineering of hyperbolic metafilm patterns. *Scientific Reports* **4**, 4498 (2014).

Note 2: SHG with 2D TP-HMMs

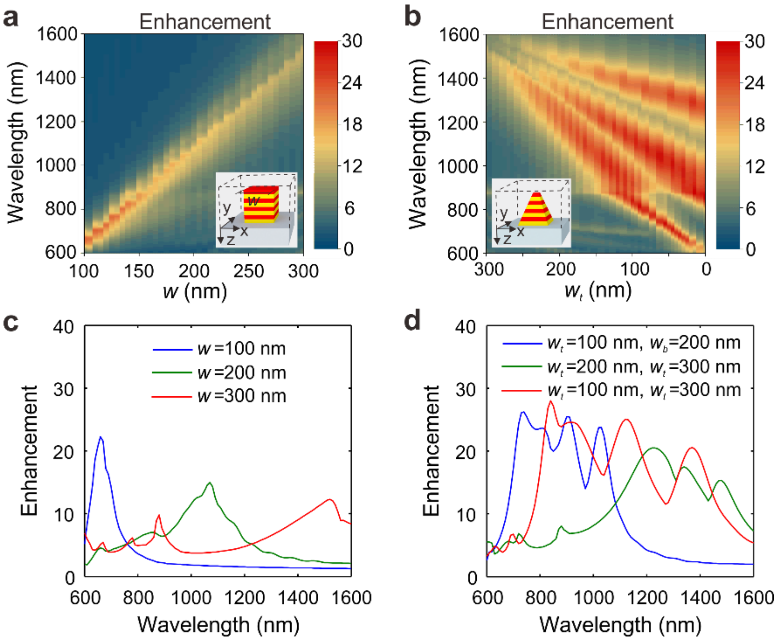

Fig. S5 Near-field enhancement. **a** Near-field enhancement of $|E_z|$ for the 2D uniform HMM with a varying *w*. **b** Near-field enhancement of $|E_z|$ for the 2D TP-HMM with a varying w_t and a constant w_b of 300 nm. **c** Near-field enhancement for the 2D uniform HMM versus the wavelength. **d** Near-field enhancement of $|E_z|$ for the 2D TP-HMM with different w_t and w_b versus the wavelength.

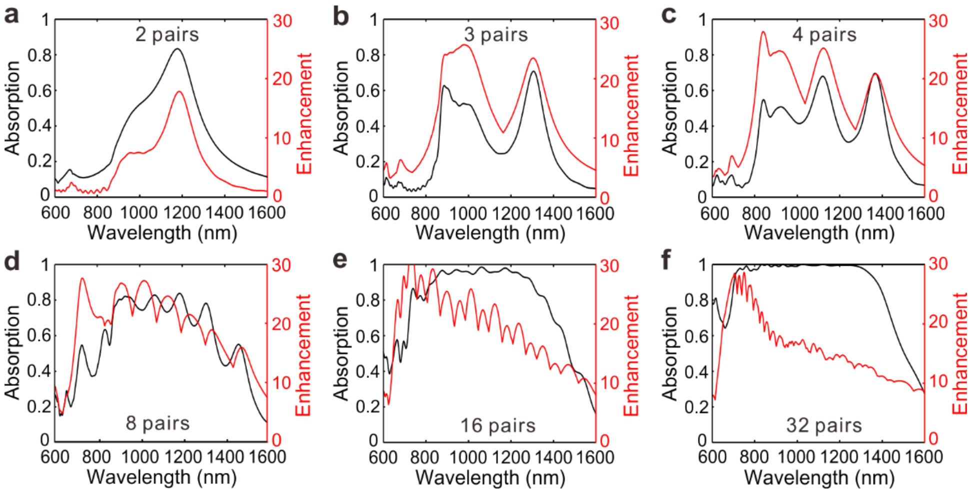

Fig. S6 Absorption and near-field enhancement of E_z for 2D TP-HMMs with varying pairs of Au/ZnO layers. The number of Au/ZnO pairs is 2 (a), 3 (b), 4 (c), 8 (d), 16 (e), and 32 (f), respectively.

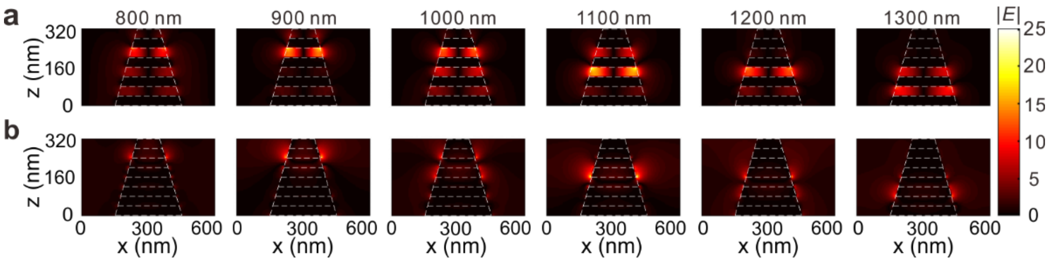

Fig. S7 Field distributions in 2D TP-HMM at the pump wavelengths to be tested. Field distributions of $|E_z|$ (a) and $|E_x|$ (b) (normalized to the incident light) in the x-z plane across the center of the 2D TP-HMM. The incident light is x-polarized and normally incident with the wavelength of 800, 900, 1000, 1100, 1200, and 1300 nm, respectively.

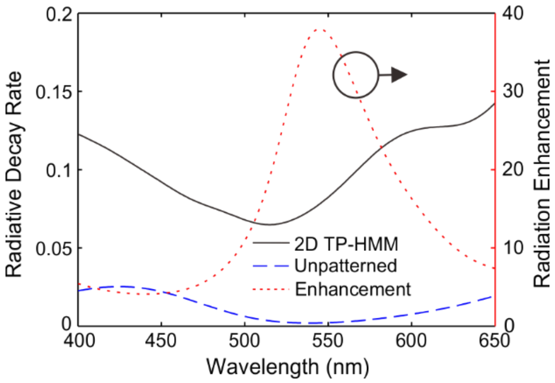

Fig. S8 Radiative decay rate and radiation enhancement. An electric dipole in the z direction is placed above the center of an array of 11×11 tapers in the simulation for the 2D TP-HMM. The radiative decay rate is obtained by the power transmission of a 2D monitor placed 500 nm above the dipole, divided by the power transmission of six monitors which form a 40×40×40 nm box around the dipole. The radiation enhancement is defined as the ratio of the radiative decay rates of the 2D TP-HMM and the unpatterned HMMs.

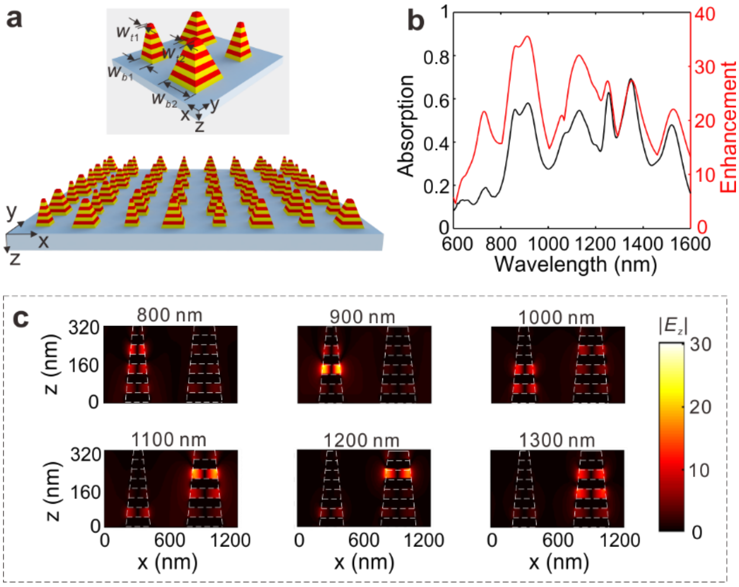

Fig. S9 Rainbow trapping in the 2D dual-pillar TP-HMM. a Schematic of the 2D dual-pillar TP-HMM, with its unit cell being shown in the upper panel. **b** Absorption and field enhancement of the dual-pillar TP-HMM. **c** Field distributions of $|E_z|$ (normalized to the incident light) in the x-z plane across the center of the 2D dual-pillar TP-HMM. The geometrical parameters are set at $w_{t1} = 80$ nm, $w_{b1} = 220$ nm, $w_{t2} = 180$ nm, and $w_{b2} = 320$ nm. The incident light is x-polarized and normally incident with the wavelengths of 800, 900, 1000, 1100, 1200, and 1300 nm, respectively.

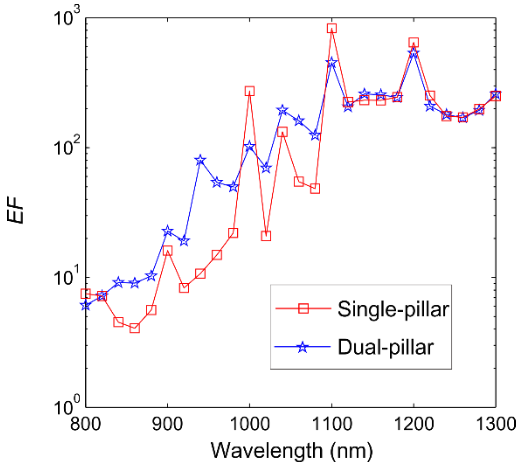

Fig. S10 Measured SHG Enhancement for the 2D TP-HMMs. *EF* for the 2D TP-HMM samples as a function of the pump wavelength under x-polarized normal incidence.

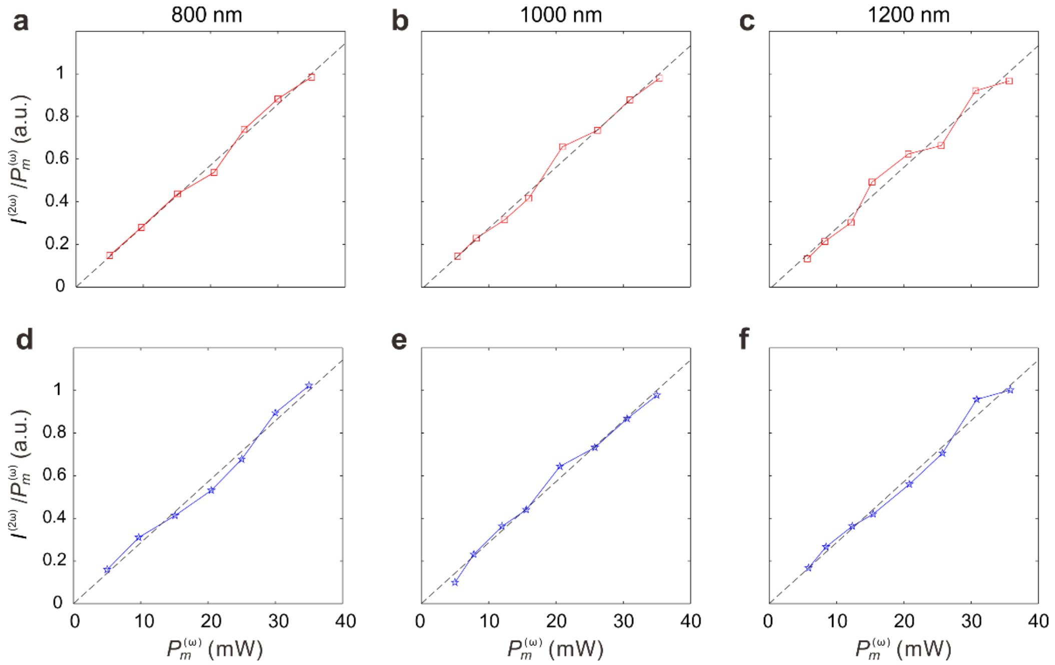

Fig. S11 Measured pump power responses for the 2D TP-HMMs. Normalized $I^{(2\omega)}/P_m^{(\omega)}$ for the 2D single-pilar (a-b) and dual-pillar (d-f) TP-HMMs as a function of $P_m^{(\omega)}$ with a pump wavelength of 800 (a, d), 1000 (b, e), and 1200 nm (c, f), respectively. The black dashed lines are the fitting data.

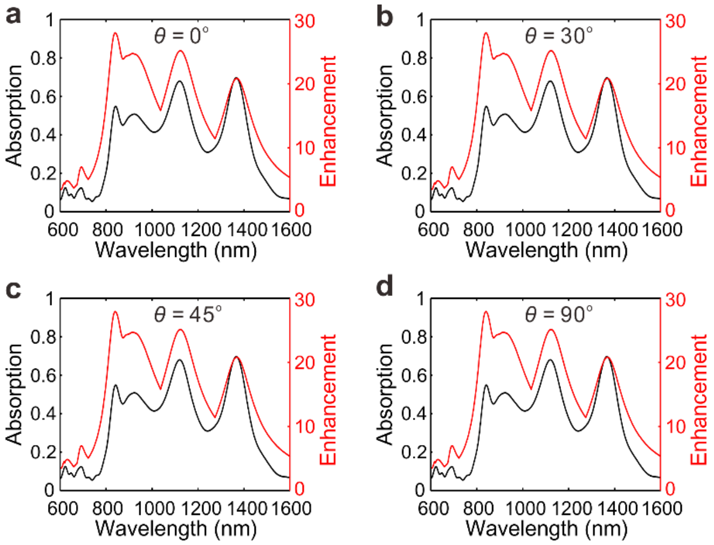

Fig. S12 Absorption and near-field enhancement of E_z for different θ. The polarization angle of the normal incidence is 0° (a), 30° (b), 45° (c), and 90° (d), respectively.

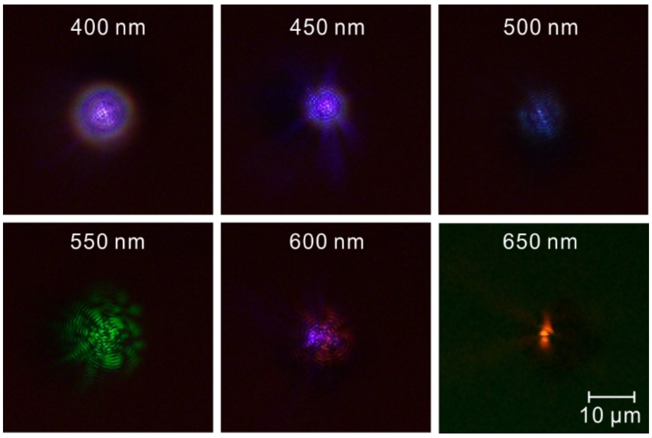

Fig. S13 Micrographs of the SHG signals for the 2D dual-pillar TP-HMM. The pump wavelength is 800, 900, 1000, 1100, 1200, and 1300 nm, respectively.

Note 3: SHG conversion efficiency

Table S1. Parameters for SHG conversion efficiency.

Pump wavelength (nm)	800	900	1000	1100	1200	1300
$R_d^{(\omega)}$	0.996	0.996	0.995	0.986	0.627	0.420
$T_o^{(\omega)}$	0.820	0.680	0.550	0.450	0.370	0.347
$P^{(\omega)}$ (mW)	10.6	8.80	7.11	5.78	3.01	1.89
SHG wavelength (nm)	400	450	500	550	600	650
$T_o^{(2\omega)}$	0.900	0.905	0.915	0.920	0.915	0.910
$T_d^{(2\omega)}$	0.948	0.968	0.970	0.970	0.970	0.970
$T_f^{(2\omega)}$	0.422	0.110	0.629	0.818	0.847	0.878
$S^{(2\omega)}$	0.463	0.809	0.975	0.980	0.941	0.848
$N^{(2\omega)}$ ($\times10^6$)	0.417	0.323	0.187	0.347	0.227	0.128
$P^{(2\omega)}$ ($\times10^{-6}$ mW)	5.96	9.91	0.820	1.16	0.766	0.464
$\eta^{(2\omega)}$ ($\times10^{-6}$)	0.560	1.13	0.115	0.200	0.255	0.245

The pump power, $P_m^{(\omega)}$, is measured by placing a light power meter (Coherent, FieldMax II-TO) between the half-wave plate and the dichroic mirror. $P_m^{(\omega)}$ is kept constant at 13 mW by adjusting the attenuator. The pump light is reflected by the short-pass dichroic mirror (Thorlabs, DMSP750B, a reflectivity of $R_d^{(\omega)}$), and transmitted through an objective (Olympus, LUCPLFLN40X, NA=0.6, a transmittance of $T_o^{(\omega)}$) before it is focused onto the samples. The power for the impinging light on the sample, $P^{(\omega)}$, can be written as

$$P^{(\omega)} = P_m^{(\omega)} R_d^{(\omega)} T_o^{(\omega)} \tag{S3}$$

The SHG signals generated by the sample is collected by the same objective with a transmittance of $T_o^{(2\omega)}$, and filtered by the short-pass dichroic mirror and the short-pass filter (Thorlabs, FES0700) with a transmittance of $T_d^{(2\omega)}$ and $T_f^{(2\omega)}$, respectively. $N^{(2\omega)}$ is retrieved by summing the intensities for all pixels in the SHG photographs (Fig. 5 in the main text). To find the mathematic relationship between $N^{(2\omega)}$ and $P^{(2\omega)}$, a 532 nm laser beam with a power of 400 mW is attenuated by 80 dB ($P_l = 4\times10^{-6}$ mW) and then photographed by the same CCD camera (Olympus, DP22). The summation of the intensities for all the pixels in the photograph of the laser beam is denoted as $N^{(532)}$ (=1.66×10^6).

The CCD spectral sensitivity is defined as, $S = CN/P$, where C is constant for different wavelengths, N and P are the corresponding total intensities for all pixels in the photograph and the light power, respectively. Then we have $P^{(2\omega)}T_o^{(2\omega)}T_d^{(2\omega)}T_f^{(2\omega)}S^{(2\omega)}/N^{(2\omega)} = P^{(532)}S^{(532)}/N^{(532)}$, where $S^{(2\omega)}$ and $S^{(532)}$ (=0.99) are the normalized CCD sensitivities at the SHG frequency and 532 nm, respectively, yielding

$$P^{(2\omega)} = \frac{N^{(2\omega)}P^{(532)}S^{(532)}}{T_o^{(2\omega)}T_d^{(2\omega)}T_f^{(2\omega)}S^{(2\omega)}N^{(532)}} \tag{S4}$$

$\eta^{(2\omega)}$ can be extracted by substituting Equations S3 and S4 into $\eta^{(2\omega)} = P^{(2\omega)}/P^{(\omega)}$

$$\eta^{(2\omega)} = \frac{N^{(2\omega)}P^{(532)}S^{(532)}}{P_m^{(\omega)}R_d^{(\omega)}T_o^{(\omega)}T_o^{(2\omega)}T_d^{(2\omega)}T_f^{(2\omega)}S^{(2\omega)}N^{(532)}} \tag{S5}$$

All the parameters for the fundamental and SHG wavelengths involved in Equation S5 are presented in Table S1.
